# Single-cell RNA sequencing uncovers an immunosuppressive program in PDAC mast cells: tumour-driven activation, proliferation, and association with Treg recruitment

**DOI:** 10.1080/07853890.2026.2724762

**Published:** 2026-08-31

**Authors:** Mengli Wu, XuXia Ye, Qi Yu, Hui Huang, Zhezhong Zhang, Xiaoli Yu, Songmei Lou, Jian Xu

**Affiliations:** ^a^ School of Medical Technology and Information Engineering, Zhejiang Chinese Medical University, Hangzhou, Zhejiang, PR China; ^b^ Department of Clinical Laboratory, The First Affiliated Hospital of Zhejiang Chinese Medical University, Hangzhou, Zhejiang, P.R. China; ^c^ Department of Pathology, Sir Run Run Shaw Hospital, School of Medicine, Zhejiang University, Hangzhou, Zhejiang, PR China; ^d^ Department of General Surgery, Sir Run Run Shaw Hospital, School of Medicine, Zhejiang University, Hangzhou, Zhejiang, PR China

**Keywords:** Pancreatic ductal adenocarcinoma, mast cell, tumour microenvironment, immunosuppression, regulatory T cells, MIF signaling, single-cell RNA sequencing, cell–cell communication

## Abstract

**Background:**

Pancreatic ductal adenocarcinoma (PDAC) is characterized by a highly immunosuppressive tumour microenvironment (TME), which contributes to its resistance to immunotherapy. Although mast cells (MCs) have been implicated in PDAC progression, their functional heterogeneity and candidate signaling models of immune modulation remain poorly understood.

**Methods:**

Through an integrated analysis of multiple single-cell RNA sequencing (scRNA-seq) datasets, we identified a significant enrichment of MCs in PDAC tissues and established a characteristic gene signature (TPSAB1, TPSB2, CPA3, HPGDS, KIT, LTC4S) for their precise identification.

**Results:**

Single-cell RNA-seq analysis categorized MCs in PDAC into resting, activated, and proliferating subpopulations. To functionally validate these transcriptomic predictions, *in vitro* co-culture experiments showed that pancreatic cancer cells promote the activation and proliferation of MCs. Cell–cell communication analysis suggested that activated MCs preferentially interact with regulatory T cells (Tregs) *via* the MIF–(CD74/CXCR4) signaling axis. This interaction was associated with an immunosuppressive T-cell landscape, characterized by an expanded population of Tregs exhibiting a highly activated immunosuppressive phenotype. Spatial transcriptomics and immunofluorescence validated the confirmed the spatial proximity of MCs and Tregs in PDAC tissues. Clinically, high expression of MC-Treg signature genes correlated with poor patient survival.

**Conclusions:**

Our study suggests that MCs are key orchestrators of immunosuppression in PDAC, predicted to interact with Tregs through the MIF–CD74/CXCR4 axis, offering a novel rationale for targeting the MC–Treg axis in future immunotherapeutic strategies.

## HIGHLIGHT

- Single-cell analysis defines a heterogeneous mast cell landscape in PDAC, encompassing resting, activated, and proliferating subpopulations.
- Pancreatic cancer-derived soluble factors directly drive mast cell activation and proliferation
- Mast cells may contribute to immunosuppression in PDAC, potentially interacting with Tregs through a prioritized MIF-(CD74/CXCR4) signaling axis that warrants direct experimental validation.

## 1. Introduction

Pancreatic ductal adenocarcinoma (PDAC) stands as one of the most aggressive gastrointestinal malignancies, projected to become the second leading cause of cancer-related deaths by 2030. Its dismal five-year survival rate of below 10% stems from the challenges of early detection, high invasiveness, metastatic potential, and profound resistance to conventional therapies [1–4]. While cancer immunotherapy, particularly immune checkpoint inhibitors (ICIs), has revolutionized the treatment of multiple cancers, its application in PDAC has been largely disappointing, with minimal clinical responses [5,6]. This therapeutic failure is primarily attributed to PDAC’s unique and complex tumour microenvironment (TME). The PDAC TME is notoriously highly fibrotic and immunosuppressive. It is dominated by an abundant and coordinated network of immunosuppressive cells—including myeloid-derived suppressor cells (MDSCs) [7,8], tumour-associated macrophages (TAMs) [9,10], and regulatory T cells (Tregs) [11]. These cells act in concert to establish an immune-quiescent state through multiple mechanisms, such as secreting inhibitory cytokines (e.g. TGF-β, IL-10), expressing immune checkpoint ligands, and metabolically starving effector T cells [12–14]. Consequently, while this suppressive network thrives, there is a marked paucity of infiltrating and functionally competent cytotoxic T lymphocytes (CTLs). Those CTLs that do manage to infiltrate often exhibit an exhausted or anergic phenotype [15]. This intricate cellular and molecular interplay effectively blunts anti-tumour immunity, fostering tumour progression and therapy resistance [16]. Therefore, a deeper dissection of the cellular interactome within the PDAC TME, particularly immunomodulatory cells and their mechanisms, is paramount for developing novel and effective immunotherapeutic strategies.

Mast cells (MCs), as tissue-resident sentinels of the innate immune system, are traditionally recognized for their critical roles in allergic reactions (type I hypersensitivity) and anti-pathogen defense [17,18]. Their function is largely mediated through the rapid degranulation of pre-formed inflammatory mediators—such as histamine, heparin, and various proteases—stored within cytoplasmic granules. Beyond this immediate response, MCs are capable of de novo synthesis and secretion of a broad spectrum of cytokines (e.g. TNF-α, IL-6, TGF-β) and chemokines (e.g. CCL2, CXCL8), enabling them to orchestrate prolonged immune responses and tissue remodeling processes [19]. In oncology, the role of MCs remains controversial and is best described as a ‘double-edged sword,’ with outcomes highly contingent on the tumour type, TME composition, and the specific activation state of the MCs themselves [20]. For instance, in certain contexts like oesophagal squamous cell carcinoma [21], ovarian cancer [22], and diffuse large B-cell lymphoma [23], MCs are reported to exert anti-tumour effects by producing molecules like IL-1, IL-6, and TNF-α. Conversely, in gastric cancer [24], lung cancer [25], melanoma [26] and breast cancer [27], MCs are frequently implicated in tumour promotion by facilitating angiogenesis, degrading the extracellular matrix to promote invasion, and—most critically—driving immunosuppression. Despite their documented presence in PDAC [28–30], their precise phenotypes, functional states, and mechanisms of action within this profoundly immunosuppressive TME remain poorly defined and represent a significant knowledge gap.

In PDAC, MCs are increasingly implicated in tumour promotion. This is supported by findings such as elevated serum tryptase (TPSAB1) in patients [28,29] and histopathological data linking high MC infiltration with metastasis and reduced survival [30,31]. A key limitation of these studies, however, is their treatment of MCs as a functionally homogeneous group. The reliance on conventional histopathology creates a ‘black box’ that obscures two fundamental questions: first, whether distinct, functionally specialized MC subpopulations exist in the PDAC TME, and second, the specific molecular mechanisms governing their pro-tumorigenic cross-talk with other immune cells. Therefore, the inability to delineate MC heterogeneity and their exact immunosuppressive mechanisms significantly hinders the development of precise therapeutic strategies.

Single-cell RNA sequencing (scRNA-seq) provides a powerful tool to analyze cellular heterogeneity, characterize cell states, and intercellular communication at single-cell resolution [32]. Herein, we apply scRNA-seq, complemented by robust bioinformatic analysis and further supported by multiplex immunofluorescence and spatial transcriptomics, to investigate the role of MCs in PDAC. We aim to elucidate the novel mechanisms by which MCs drive PDAC immunosuppression. Our findings are expected to provide a solid theoretical foundation for developing novel combination immunotherapies that target MCs or their key signaling pathways in PDAC.

## 2. Methods

### 2.1. Material

The study utilized various public datasets, including one Spatial Transcriptomics dataset, three scRNA-seq datasets (GSE212966, GSE155698, GSE205049), and bulk RNA-seq data including the TCGA-PAAD cohort and one Gene Expression Omnibus (GEO) microarray dataset (GSE15471). The transcriptomic and clinical data for TCGA-PAAD were obtained from UCSC Xena browser (http://xena.ucsc.edu/). Data for GEO datasets were acquired from the GEO database (https://www.ncbi.nlm.nih.gov/geo/) (Supplementary Table 1).

### 2.2. Cell culture

Human MC HMC-1 and Human pancreatic cancer cells BxPC-3 were cultured in RPMI 1640 medium (Gibco) containing 10% fetal bovine serum (FBS) (Gibco) with 100 U/mL penicillin and 100 μg/mL streptomycin. LAD2 cells were grown in StemPro-34 medium (Gibco) supplemented with 100 ng/ml stem cell factor (Novoprotein), 100 μg/ml IL-6 (Novoprotein), nutrient supplement (Gibco), 100 U/ml penicillin (Invitrogen), 100 µg/ml of streptomycin (Invitrogen) and 2 mM L-Glutamine (Gibco). Human pancreatic cancer cell PANC-1 and Human pancreatic ductal epithelial cells HPNE were cultured in DMEM medium (Gibco) containing 10% FBS (Gibco) with 100 U/mL penicillin and 100 μg/mL streptomycin.

### 2.3. Total RNA extraction and qRT-PCR

Human tissue samples were obtained from Sir Run Run Shaw Hospital with approval from the Institutional Review Board. Total RNA was extracted from tumour tissue and matched adjacent non-cancerous tissue from 5 PDAC patients using FreeZol Reagent (Vazyme, Nanjing, Jiangsu, China). The obtained RNA was reverse transcribed into cDNA using HiFiScript All-in-one RT Master Mix for qPCR (CWBIO, Beijing, China). Subsequently, the expression levels of CPA3, HPGDS, KIT, LTC4S, TPSAB1, and TPSB2 were measured using qRT-PCR with SuperStar Universal SYBR Master Mix (CWBIO, Beijing, China). GAPDH was used as an internal control. The relative mRNA expression was calculated using the 2^−ΔΔCt^ method. The primer sequences are provided at supplementary table (Supplementary Table 3). Each assay was performed in three biological replicates.

### 2.4. Immunofluorescence staining

Human tissue specimens were obtained from Sir Run Run Shaw Hospital between 2024 and 2025 with the approval of the Institutional Review Board. Immunofluorescence staining was performed on tumour tissue sections from 5 PDAC patients (Supplementary Table 2). The tissue sections were placed in an 80 °C water bath for antigen retrieval for 20–30 min. Afterward, the sections were allowed to cool to room temperature, followed by washing with PBS for 5 min each time, repeated three times. To block nonspecific binding, the sections were incubated with QuickBlock Immunostaining Blocking Solution (Beyotime, Shanghai, China) for 2 h. Subsequently, the sections were incubated with primary antibodies overnight at 4 °C in a humidified chamber. The primary antibodies used to validate MCs and Treg cells included rabbit anti-human KIT mAb (ABclonal, Cat# A27823, 1:150), rabbit anti-human TPSAB1 mAb (LSBio, Cat# LS-C482340-30, 1:100) and mouse anti-human Foxp3 mAb (Abcam, Cat# ab20034, 1:500). After thorough washing, the sections were incubated with fluorescently labeled secondary antibodies: ABflo^®^ 488-conjugated Goat anti-Mouse IgG (H + L) (ABclonal, Cat# AS076, 1:150) and ABflo^®^ 647-conjugated Goat anti-Rabbit IgG (H + L) (ABclonal, Cat# AS060, 1:150). Following three additional PBS washes, nuclei were stained with DAPI, and slides were mounted with anti-fade mounting medium (Beyotime, Cat# P0126). Imaging was performed using a NIKON ECLIPSE CI microscope, and images were analyzed with CaseViewer software.

### 2.5. Toluidine blue staining

Human tissue specimens were obtained from Sir Run Run Shaw Hospital between 2024 and 2025 with the approval of the Institutional Review Board. Tumour tissue and matched adjacent non-cancerous tissue from 5 PDAC patients were collected and sectioned for toluidine blue staining (Supplementary Table 2). Tissue sections were deparaffinized by sequential immersion in xylene (10 min, twice), followed by a graded ethanol series (100%, 100%, 95%, 80%; 5 min each) and PBS (5 min). Sections were stained in 0.1% toluidine blue solution for 1 h, rinsed briefly in water, and differentiated in 95% ethanol for 5 s. Imaging was performed using a NIKON ECLIPSE CI microscope, and images were analyzed with CaseViewer software.

### 2.6. HMC-1 and LAD2 cell degranulation

The indicated MCs and pancreatic cancer cells were directly co-cultured in the same well of a six-well plate. For all cell-line combinations, including HMC-1 + PANC-1 and LAD2 + BxPC-3, 3 × 10^6^ MCs and 3 × 10^5^ pancreatic cancer cells were cultured in a total volume of 2 mL per well, consisting of 1 mL of MC culture medium and 1 mL of pancreatic cancer-cell culture medium. The cells were directly co-cultured for 48 h at 37 °C in a humidified atmosphere containing 5% CO_2_. The corresponding control cells were cultured separately for the same duration under identical conditions, with the same culture medium composition and total volume as the co-culture group. After co-culture, the indicated samples were collected for subsequent downstream analyses.

PANC-1 cell and HPNE were co-cultured with HMC-1 cells for 48 h. BxPC3 cell was co-cultured with LAD2 cells for 48 h. Subsequently, MCs and the culture supernatant were collected. Citrate substrate was then added, followed by gentle tapping for thorough mixing. After incubation in the dark, the stop solution was added. The absorbance was measured at a wavelength of 405 nm using a microplate reader. The degree of MCs degranulation was determined by detecting the release of β-hexosaminidase.

### 2.7. Cell cycle

After MCs were cultured with pancreatic cancer cell for 48 h, the cells were washed with phosphate-buffered saline (PBS). Subsequent operations were performed strictly in accordance with the instructions of the Cell Cycle and Apoptosis Analysis Kit (Beyotime, Cat# C1052). Finally, a flow cytometer was used to detect and analyze the cell cycle distribution of HMC-1 cells in each group.

### 2.8. Single-cell sequencing data processing

Each scRNA-seq dataset was processed independently using Seurat, and no cross-dataset integration was performed. Cells from different datasets were not merged for joint dimensionality reduction or clustering. For each dataset, low-quality cells were excluded according to the following criteria: cells with fewer than 200 or more than 10,000 detected genes, cells with a total unique molecular identifier count exceeding 30,000, or cells with a mitochondrial transcript proportion greater than 25%.

After quality-control filtering, an initial SCTransform-based preprocessing procedure was performed within each dataset to support doublet identification. SCTransform (v 0.4.1) was used for normalization and variance stabilization, with sequencing depth modeled internally and mitochondrial transcript proportion included as a technical covariate. Principal component analysis was subsequently performed using the SCTransform-normalized expression matrix. Potential doublets were identified separately in each dataset using DoubletFinder. The expected doublet rate was determined according to the number of recovered cells in each dataset, and cells classified as doublets were excluded from subsequent analyses.

Following doublet removal, SCTransform normalization and principal component analysis were repeated using the retained singlet cells. No additional LogNormalize, NormalizeData, FindVariableFeatures, or ScaleData workflow was applied in the final analysis. Furthermore, no explicit batch-correction or integration algorithm, including Seurat CCA, RPCA, or Harmony, was applied either across datasets or among samples within an individual dataset.

For major cell-type identification, a shared nearest-neighbour graph was constructed separately for each dataset using the first 50 principal components with the FindNeighbors function. Cell clusters were identified using FindClusters at a resolution of 0.8. t-distributed stochastic neighbour embedding was used only for visualization of the independently generated clustering results. Cell types were annotated according to the expression of established lineage-specific marker genes.

To further characterize MC and T-cell heterogeneity, MCs and T cells were extracted from each dataset and reanalyzed separately. For these subset analyses, the neighbour graph and clustering were generated using the first 20 principal components, with a clustering resolution of 1.0. All downstream analyses were performed separately for each dataset.

Cross-dataset comparisons were based on independently derived results rather than an integrated cell-level representation. Specifically, we evaluated the consistency of cell-type and subpopulation annotations, marker-gene expression patterns, functional signatures, and sample-level cell proportions across datasets. Statistical analyses of cell proportions were conducted using individual biological samples, rather than individual cells, as the unit of analysis. Prior to downstream analyses, potential doublets were identified and removed using DoubletFinder [33]. Default parameters were applied, and the estimated doublet rate was adjusted based on the number of cells in each dataset. This approach allowed for further characterization of cellular heterogeneity and subpopulation features.

### 2.9. Expression difference analysis

Differentially expressed genes (DEGs) for each cell cluster or subset were identified using the ‘FindAllMarkers’ function in Seurat. Genes were considered as MC marker genes in major cell populations if they met the following criteria: log fold-change of average expression > 1, pct.1 (percentage of expressed cells in MCs) > 0.7, pct.2 (percentage of expressed cells in other cells) < 0.3, and adjusted P value < 0.01. Differential-expression analysis was primarily used for exploratory characterization and identification of transcriptional features of cell populations. We acknowledge that cell-level differential-expression analysis may not fully account for biological variability across individual donors in multi-sample scRNA-seq datasets, which represents a limitation of the current study.

### 2.10. Bioinformatics analysis

**Trajectory analysis:** Pseudotime trajectories of MC subsets were constructed using the Monocle 2 package (version 2.28.0). The DDRTree method implemented with the ‘reduceDimension’ function of Monocle 2 was used for dimensionality Reduction and construction of pseudo-temporal order. **Cellular interaction analysis by CellChat:** Ligand-receptor communication networks were inferred using CellChat (v1.6.1) for the GSE212966 dataset. Ligands and receptors were considered expressed if present in ≥10% of cells in the corresponding cluster. Interactions observed in fewer than 10 cells were removed using filterCommunication(min.cells = 10) [34]. Communication probabilities were computed with computeCommunProb(raw.use = TRUE, population.size = TRUE), followed by pathway-level aggregation and network analysis using standard CellChat functions. We acknowledge that intercellular communication prediction is method-dependent, and that different frameworks (e.g. CellPhoneDB, NATMI, ICELLNET) may yield complementary results. **Functional and Pathway Enrichment Analysis:** Gene Ontology Biological Process (GO BP) enrichment analysis of DEGs was performed using the Xiantao Academic Platform (https://www.xiantaozi.com/). **Correlation Analysis** *via* **the BEST Platform:** Correlations between MC markers (TPSAB1, TPSB2, HPGDS, KIT, CPA3) and Th1/Treg cell signatures were assessed using the BEST online tool (https://rookieutopia.hiplot.com.cn/app_direct/BEST/). **Prognosis analysis of MC signature genes:** The MC/Treg signature score was calculated using normalized expression levels of three marker genes (KIT, IL2RA, TPSAB1). Patients were classified into ‘High’ or ‘Low’ groups according to the optimal cutoff determined by the survminer::surv_cutpoint() function. Survival differences between groups were evaluated by univariate log-rank test, and Kaplan-Meier curves were generated using the survfit function. Due to limited sample size, multivariable Cox regression was not performed, and therefore independence from other clinical factors could not be established. **CIBERSORT analysis:** Immune cell fractions in GSE15471 datasets were estimated using CIBERSORT (Moonerss version) with the LM22 signature matrix. Output provided 22 immune cell proportions per sample, which were merged with tumour/normal group information. Boxplots and barplots were generated in R (ggplot2, ggpubr). Statistical differences between groups were tested using Wilcoxon rank-sum test (*p < 0.05). **GSVA:** GSVA was performed using the GSVA R package (v1.44.0). Hallmark gene sets were obtained from MSigDB. Expression data from MCs (Activated MC and Resting MC) were extracted from the Seurat object (GetAssayData) and converted to a matrix. GSVA scores were calculated using a Gaussian kernel, and scores were stored in a new Seurat object with metadata. Differential pathway activity between Activated MC and Resting MC was evaluated using limma, applying linear modeling (lmFit) and empirical Bayes moderation (eBayes). *p*-values were adjusted using the Benjamini–Hochberg (BH) method. **GEPIA analysis:** Gene expression and correlation analyses were performed using the GEPIA web tool (http://gepia.cancer-pku.cn). **TIMER analysis:** Correlation between gene expression and immune cell infiltration was analyzed using TIMER2.0 (http://timer.cistrome.org). Spearman correlation coefficients were calculated, and *p* < 0.05 was considered statistically significant. **Propeller:** To assess changes in cell-type proportions at the sample level, we used the propeller method, which performs statistical testing of cell-type abundance while accounting for biological replication across patients [35]. For each single-cell dataset, we aggregated cell counts by sample and applied propeller to test for significant differences in cell-type proportions.

### 2.11. Spatial transcriptomic analysis

Spatial transcriptomic data from the 7 PDAC samples (GSE235315) were normalized using the ‘SCTransform’ function in the ‘Seurat’ package. Tumour and adjacent normal regions were annotated based on histological features and H&E images. Dimensionality reduction and clustering were performed using ‘RunPCA’ and ‘RunUMAP’ (with 15 principal components and a resolution of 0.8). After merging similar clusters, we identified normal and tumour regions in the PDAC samples. To assess the spatial distribution of the MC activation signature, we utilized the ‘AddModuleScore’ function in the ‘Seurat’ package.

### 2.12. Statistical analysis

All statistical analyses were performed using R (version 4.2.2) and GraphPad Prism (version 9). Differences between two groups were compared using Student’s t-test. Survival analyses employed univariate Cox regression and the Log-Rank methods. A *p*-value <0.05 was considered statistically significant. The exact statistical test, number of independent biological replicates, and definition of error bars for each analysis are provided in the corresponding figure legends.

## 3. Result

### 3.1. Specific enrichment of MCs in PDAC and identification of their Characteristic gene signature

**Figure 1.**
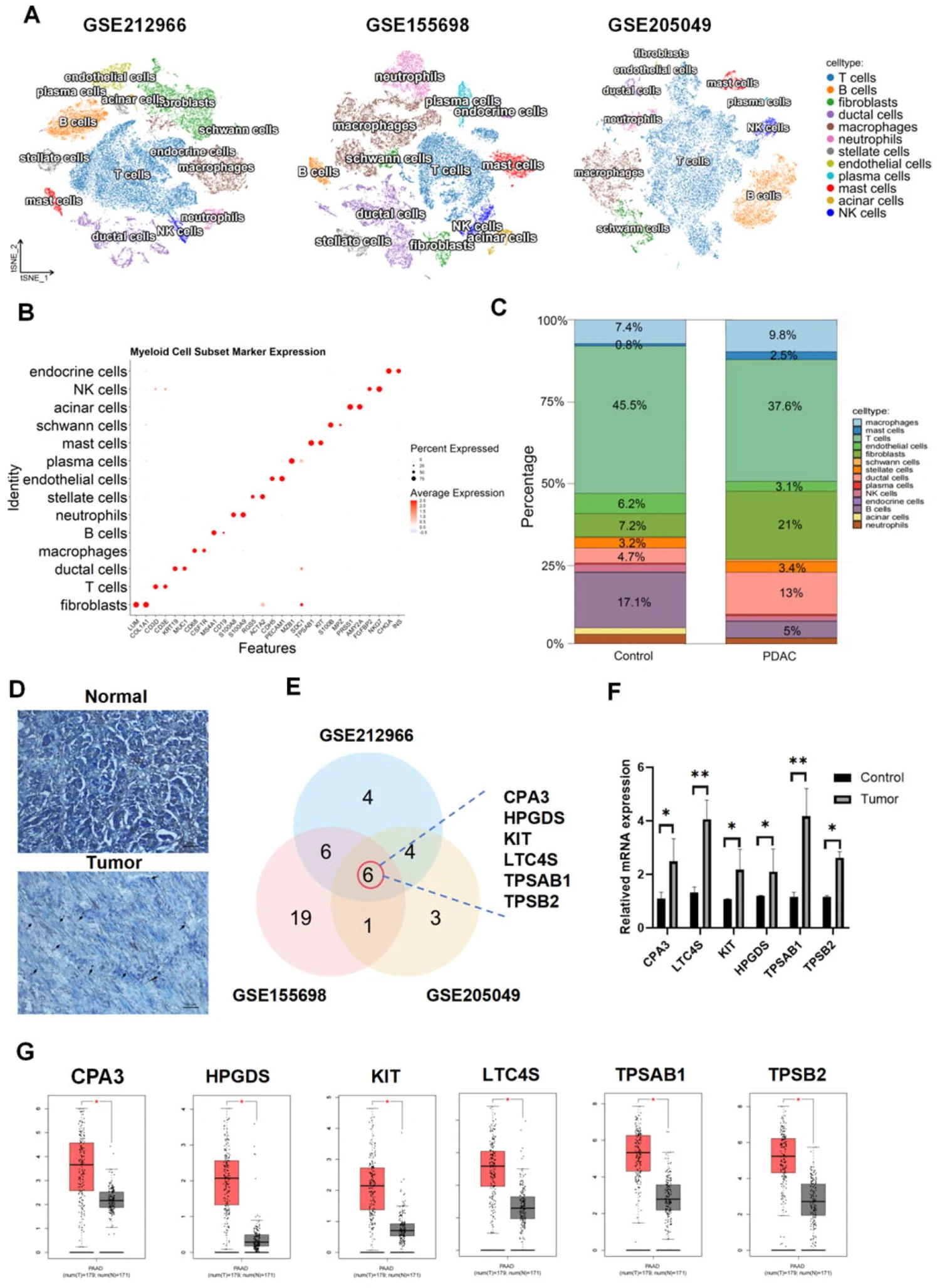
Specific Enrichment of MCs in PDAC and Identification of Their Characteristic Gene Signature. (A) UMAP plots displaying the major cell types in the GSE212966, GSE155698, and GSE205049 datasets. (B) Dot plots of marker genes for each major cell type. (C) Cell proportion plots of each major cell type in PDAC and adjacent non-tumour tissues across. (D) Toluidine blue staining images showing the number of MCs in PDAC and adjacent non-tumour tissues. (E) Venn diagrams (centre) of differentially expressed genes (DEGs) in MCs in the GSE212966, GSE155698, and GSE205049 datasets, with the intersection showing the six MC signature genes. The screening DEGs are depicted within the dotted lines. (F) qRT-PCR analysis shows the expression levels of six MC signature genes in PDAC and adjacent non-tumour tissues. Data are shown as mean ± SEM (n = 5 biological replicates). Statistical significance was determined by two-tailed Student’s t-test. *p < 0.05, **p < 0.01. Each bar represents the mean of the biological replicates. (G) GEPIA web portal analysis shows the expression levels of six MC signature genes in pancreatic cancer tissues and normal tissues.

To comprehensively characterize the cellular landscape of the PDAC TME, we integrated and analyzed three independent scRNA-seq datasets (GSE212966, GSE155698, GSE205049). We performed stringent quality control by filtering out low-quality cells based on the number of genes, UMIs, and mitochondrial gene content to guarantee the reliability of all downstream analyses (Figure S1A, B). First, we annotated major clusters based on defined marker genes and identified various cell types, including fibroblasts (LUM, COL1A1), T cells (CD3D, CD3E), ductal cells (KRT19, MUC1), macrophages (CD68, CSF1R), B cells (MS4A1, CD19), neutrophils (S100A8, S100A9), stellate cells (RGS5, ACTA2), endothelial cells (CDH5, PECAM1), plasma cells (MZB1, SDC1), MCs (TPSAB1, KIT), Schwann cells (S100B, MPZ), acinar cells (PRSS1, AMY2A), NK cells (FGFBP2, NKG7), and endocrine cells (CHGA, INS) (Figure 1A, B). Comparative analysis revealed a significant enrichment of MCs in tumour tissues compared to adjacent normal tissues (Figure 1C). This finding was further supported by sample-level analysis using the propeller method, confirming that the enrichment is statistically significant across individual patient samples rather than being driven by pooled cell percentages (Figure S5). This finding was corroborated by toluidine blue staining of clinical specimens, which confirmed the increased MC infiltration in PDAC lesions (Figure 1D).

To precisely define the MC population, we first annotated MCs using canonical markers TPSAB1 and KIT across all single-cell datasets. For each dataset, we then isolated MCs from tumour and paired adjacent non-tumour tissues and performed differential expression analysis. Genes were considered significantly upregulated in tumour MCs if they met the following criteria: log2 fold change > 1, expression proportion in MCs (PCT1) > 0.7, expression proportion in other cell types (PCT2) < 0.3, and *P*-value < 0.01. Applying this workflow across all three datasets, we consistently identified six core genes—TPSAB1, TPSB2, CPA3, HPGDS, KIT, and LTC4S as a characteristic gene signature for PDAC MCs (Figure 1E). Because these comparisons did not account for the non-independence of cells originating from the same donor, the resulting expression differences are presented as descriptive, hypothesis-generating cell-state patterns rather than confirmatory donor-level differential-expression findings. We further examined the expression of these six signature genes using the GEPIA database (Figure 1G) and clinical samples (Figure 1F). The higher expression observed in PDAC tissues is consistent with increased MC abundance or activation, supporting our single-cell findings. The results indicated that all six genes were significantly more highly expressed in tumour tissues. In summary, our results demonstrate the specific enrichment of MCs in PDAC and define a reliable gene signature for their identification.

### 3.2. Activation and functional heterogeneity of MCs in the PDAC microenvironment

**Figure 2.**
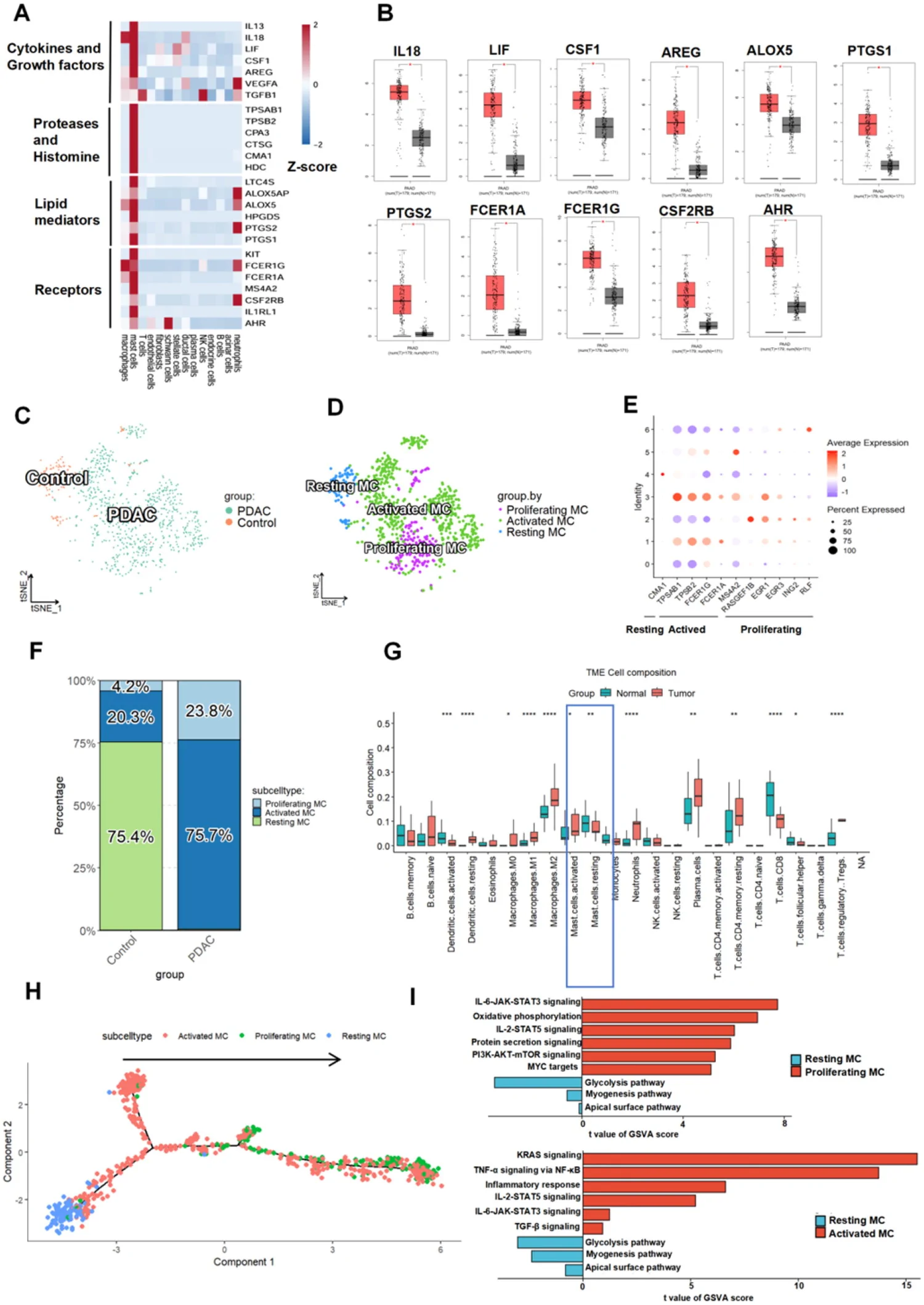
Activation and Functional Heterogeneity of MCs in the PDAC Microenvironment. (A) Heatmap showing the expression of cytokine and growth factor, protease and histamine, lipid mediator, and various receptor-related genes across different major cell types. (B) GEPIA web portal analysis shows the expression profiles of cytokine and growth factor-related genes, protease and histamine-related genes, lipid mediator-related genes, and various receptor-related genes across PDAC and normal tissues. (C) UMAP plots of MCs coloured by tissue type. (D) UMAP plots of MCs coloured by cluster. (E) Dot plots of marker genes for MCs subtypes. (F) Bar charts show the proportion of MC subtypes. (G) CIBERSORT shows the difference in the proportion of activated and resting MCs between Normal tissue and PDAC. (H) Differentiation trajectory of MCs with each colour coded for MC subsets. (I) Differential pathways enriched in activated MCs and proliferating MCs by GSVA, showing the top 9 significantly enriched hallmark terms.

To investigate the activation status of MCs at single-cell resolution, we first analyzed the expression of MC activation-related receptors and mediators. Our results demonstrated that PDAC MCs highly expressed a repertoire of activation-related receptors, including KIT, the complete high-affinity IgE receptor subunits (FCER1A, MS4A2, FCER1G), as well as CSF2RB, IL1RL1, and AHR. Concurrently, these MCs exhibited elevated expression of hallmark proteases (TPSAB1, TPSB2, and CPA3) and key enzymes involved in the biosynthesis of inflammatory mediators, such as histamine (HDC), leukotrienes (LTC4S, ALOX5), and prostaglandins (HPGDS, PTGS1), and MCs served as a significant source of various cytokines and growth factors, including IL13, LIF, CSF1, AREG, IL18, and VEGFA (Figure 2A). Furthermore, we also confirmed significantly higher expression of these critical activation-related molecules in pancreatic cancer versus normal tissues *via* the GEPIA database (TCGA data) (Figure 2B). Given the overall activated state of MCs in PDAC, we next sought to deconvolute their functional heterogeneity. We performed unsupervised clustering on the MC compartment, which identified three distinct subpopulations: resting MCs (characterized by CMA1), activated MCs (characterized by TPSAB1/TPSB2 and FCER1G/MS4A2), and proliferating MCs (characterized by MKI67 and TOP2A) (Figure 2C–E). Cellular proportion analysis revealed that the proportions of both activated and proliferating MC subpopulations were significantly increased in PDAC tissue, whereas the proportion of resting MCs was significantly decreased. (Figure 2F). This finding was further supported by sample-level analysis using the propeller method, confirming that the enrichment is statistically significant across individual patient samples rather than being driven by pooled cell percentages (Figure S6). This finding was consistent with the CIBERSORT analysis of bulk transcriptomic data (GSE15471), which also indicated an increased proportion of activated MCs and a decreased proportion of resting MCs (Figure 2G). To explore the relationships between these subpopulations, we performed pseudotime trajectory analysis, which illustrated an inferred trajectory or state continuum of MCs encompassing resting, activated, and proliferating states (Figure 2H).

To functionally characterize these subpopulations, we performed differential expression analysis. Compared to resting MCs, activated MCs exhibited significant up-regulation of 225 genes, including key effector molecules such as (e.g. TPSAB1, HSPA1A), and down-regulation of 31 genes (Figure S2A); proliferating MC exhibited significant up-regulation of 41 genes, including key effector molecules such as (e.g. FOSB, JUN), and down-regulation of 35 genes (Figure S2B). Gene Set Variation Analysis (GSVA) further uncovered subpopulation-specific pathway enrichments. Activated MCs were significantly enriched in the KRAS signaling pathway, TNF-α signaling *via* NF-κB pathway, inflammation response and so on, aligning with core oncogenic drives and the inflammatory microenvironment of PDAC. Proliferating MCs specifically activated pathways such as IL-6/JAK/STAT3 signaling pathway, IL-2-STAT5 signaling pathway, PI3K/AKT/mTOR signaling pathway and so on, which are pivotal for cell proliferation and survival (Figure 2I). Collectively, based on scRNA-seq data, these results indicate that the PDAC microenvironment not only induces a robust activation of MCs but also shapes their functional heterogeneity.

### 3.3. Under the conditioning of pancreatic cancer cell-derived soluble factors, MCs exhibit a pronounced activated and proliferative phenotype

**Figure 3.**
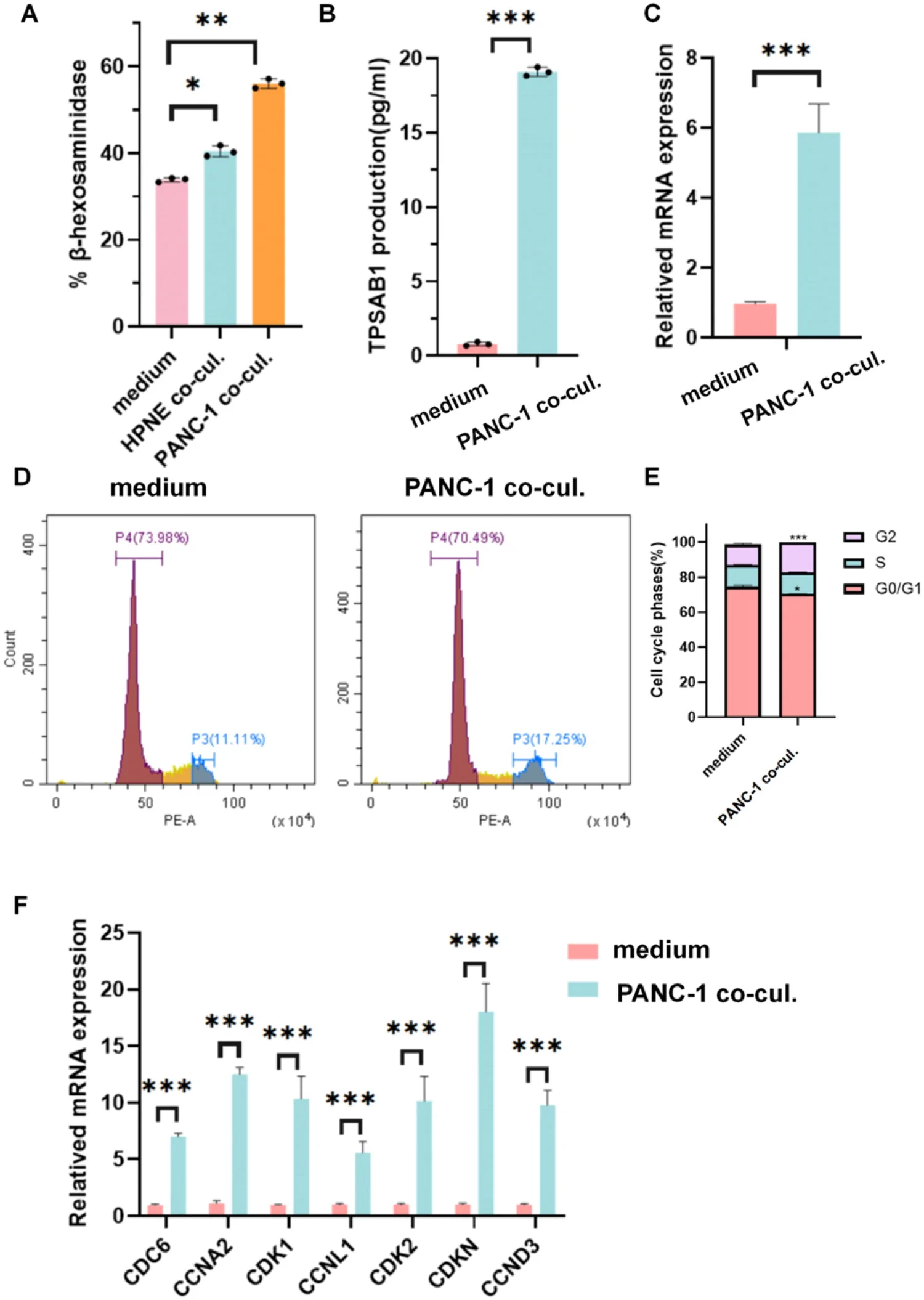
Under the conditioning of pancreatic cancer cell-derived soluble factors, MCs exhibit a pronounced activated and proliferative phenotype. (A) MCs degranulation following co-culture with HPNE and PANC-1. (B) TPSAB1 secretion level detected by ELISA following co-culture of MCs with PANC-1. (C) qRT-PCR analysis shows the expression levels of TPSAB1 in MCs after co-culture with PANC-1. (D,E) Flow cytometry analysis shows the cell cycle distribution of MCs after co-culture with PANC-1. (F) qRT-PCR analysis shows the expression levels of Cell cycle-related genes in MCs after co-culture with PANC-1. Data are shown as mean ± SEM from n = 5 biological replicates. Statistical significance was determined by two-tailed Student’s t-test. *p < 0.05, **p < 0.01, ***p < 0.001.

To validate the expansion of the activated and proliferating MC subpopulations observed in sc-RNA seq, we investigated whether soluble factors from pancreatic cancer cells could directly induce MC activation and expansion *in vitro*. Specifically, Co-culture of pancreatic cancer cells (PANC-1 co-cul.) with MCs directly induced MC degranulation, leading to a significant release of the hallmark mediators β-hexosaminidase (Figure 3A) and TPSAB1 (Figure 3B). Concurrently, a marked up-regulation of TPSAB1 gene expression was observed (Figure 3C), confirming the activation status of MCs in the pancreatic cancer context. Furthermore, co-culture with PANC-1 significantly facilitated MC proliferation (Figure 3D,E), and this pro-proliferative effect was supported by the coordinated up-regulation of key cell cycle-related genes, including CDC6, CCNA2, CDK1, CCNL1, CDK2, CDKN, and CCND3 (Figure 3F). These findings collectively indicate that the pancreatic cancer microenvironment directly orchestrates both the functional activation and expansion of the MC population. Consistently, co-culture of LAD2 with BxPC-3 cells produced similar activation and proliferative effects, confirming the generality of these observations across different MC and pancreatic cancer cell models (Figure S4).

### 3.4. Activated MCs orchestrate an immunosuppressive T-cell landscape in PDAC

**Figure 4.**
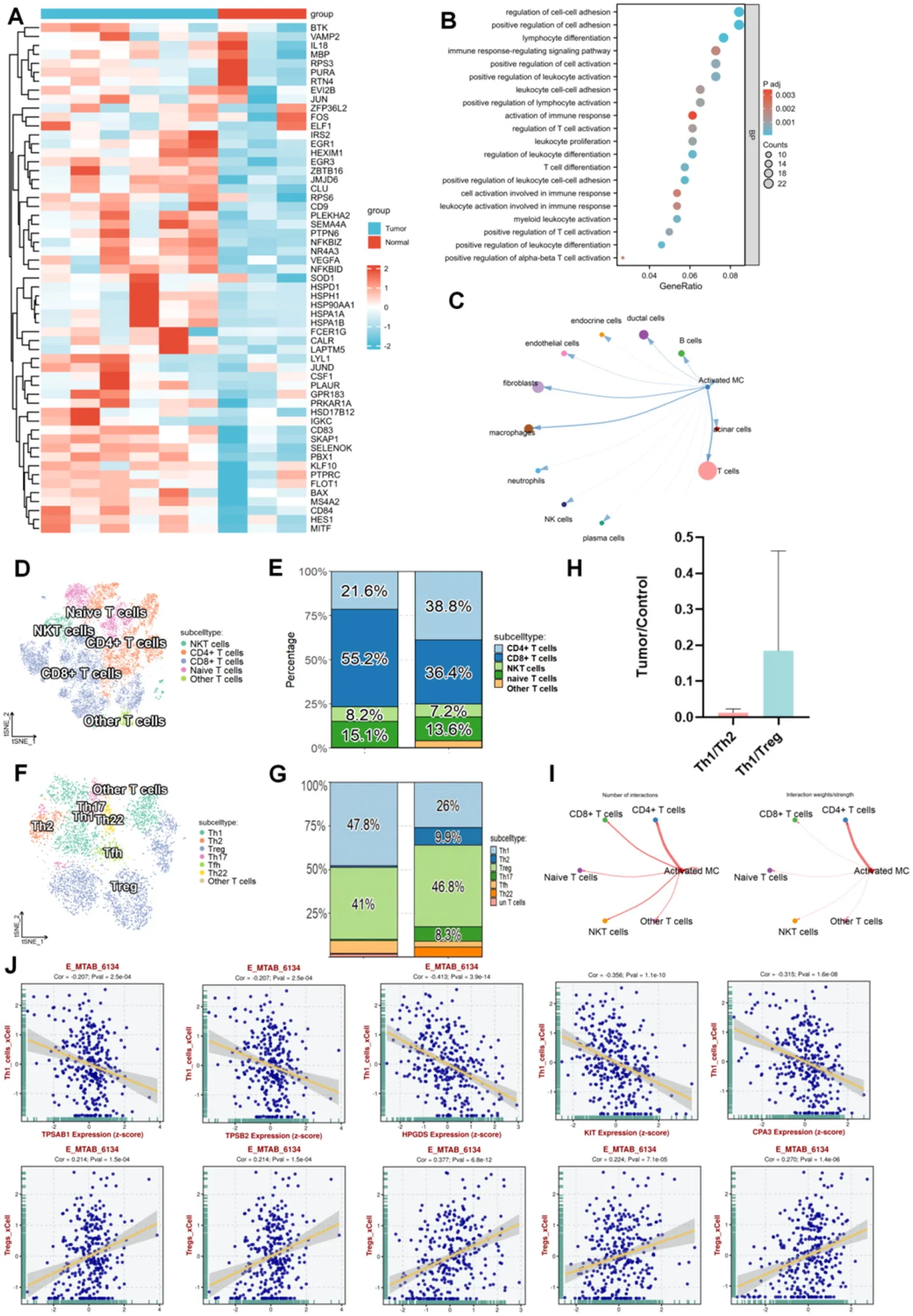
Activated MCs orchestrate an immunosuppressive T-cell landscape in PDAC. (A) Heatmap showing the DEGs of MCs in PDAC and adjacent non- tumour tissues. The tissue type is indicated by the colour above the heatmap. (B) KEGG enrichment analysis of up-regulated DEGs in PDAC MCs. (C) Chord diagram generated by CellChat analysis showing the interaction strength between activated MCs and other major cell types in PDAC. (D) UMAP plots of T cell subtypes coloured by cluster. (E) Bar charts show the proportion of T cell subtypes. (F) UMAP plots of CD4^+^ T cell subtypes coloured by cluster. (G) Bar charts show the proportion of CD4^+^ T cell subtypes. (H) Bar graph displays the relative ratios of Th1/Th2 and Th1/Treg subsets between tumour tissues and control samples (presented as Tumour/Control). (I) Chord diagram generated by CellChat analysis showing the interaction strength and the interaction number between activated MCs and T cell subtypes in PDAC. (J) Scatter plot matrix illustrates the correlations between the expression levels (x-axis, z-score normalized) of MC function-related genes (TPSAB1, TPSB2, HPGDS, KIT, CPA3) and the abundances (y-axis) of T cell subsets (Th cells, Treg cells).

To investigate the functional role of MCs in PDAC, we first analyzed the transcriptome of tumour-infiltrating MCs. Differential gene expression analysis revealed significant up-regulation of multiple genes, including BTK, IL18, CSF1, HSPD1 and so on (Figure 4A). KEGG indicated that these up-regulated genes were significantly involved in immune cell activation and regulation. Specifically, processes such as ‘positive regulation of T cell activation’, ‘lymphocyte differentiation’, and ‘regulation of cell-cell adhesion’ were highly prominent (Figure 4B), indicating that MCs are actively engaged in immunomodulatory crosstalk within the tumour microenvironment.

Based on these findings, we hypothesized that activated MCs modulate the tumour microenvironment through extensive crosstalk with immune cells. We therefore employed CellChat to map cell-cell communication networks. The results showed that the strength of cell interactions between activated MCs and T cells were among the most prominent (Figure 4C). Given the prominent interactions between MCs and T cells, we next profiled the T-cell compartment (Figure 4D; Figure S3A,S3B). We observed an overall skewing of the T-cell landscape in PDAC, characterized by an increased proportion of CD4^+^ T cells and concomitant decreases in CD8^+^ T cells, NKT cells, and naïve T cells (Figure 4E). Next, we mapped MCs communication networks with different T cell subsets. CellChat analysis revealed that among all T cells, the interactions between activated MCs and CD4^+^ T were the most prominent, exhibiting the greatest number and strength (Figure 4I). Further deep phenotyping of CD4^+^ T cells revealed a shift towards immunosuppression: the proportions of anti-tumour Th1 and T follicular helper (Tfh) cells were reduced, whereas pro-tumour subsets including Th2, Th17, Th22, and particularly immunosuppressive Tregs were enriched (Figure 4F,G; Figure S3B). This finding was further supported by sample-level analysis using the propeller method, confirming that the enrichment is statistically significant across individual patient samples rather than being driven by pooled cell percentages (Figure S7). Consistent with this skewed subset distribution, the ratios of Th1/Th2 and Th1/Treg were significantly decreased (Figure 4H). To independently validate the association between MCs and this immunosuppressive T-cell phenotype, we performed correlation analysis with the TIMER database, which confirmed a significant negative correlation between MC signature genes and Th1 cells and a strong positive correlation with Tregs (Figure 4J). These findings collectively indicate that activated MCs are associated with a highly immunosuppressive T cell landscape.

### 3.5. The MIF signaling axis may facilitate strong MC-Treg interactions and potentially contribute to Treg functional maturation

**Figure 5.**
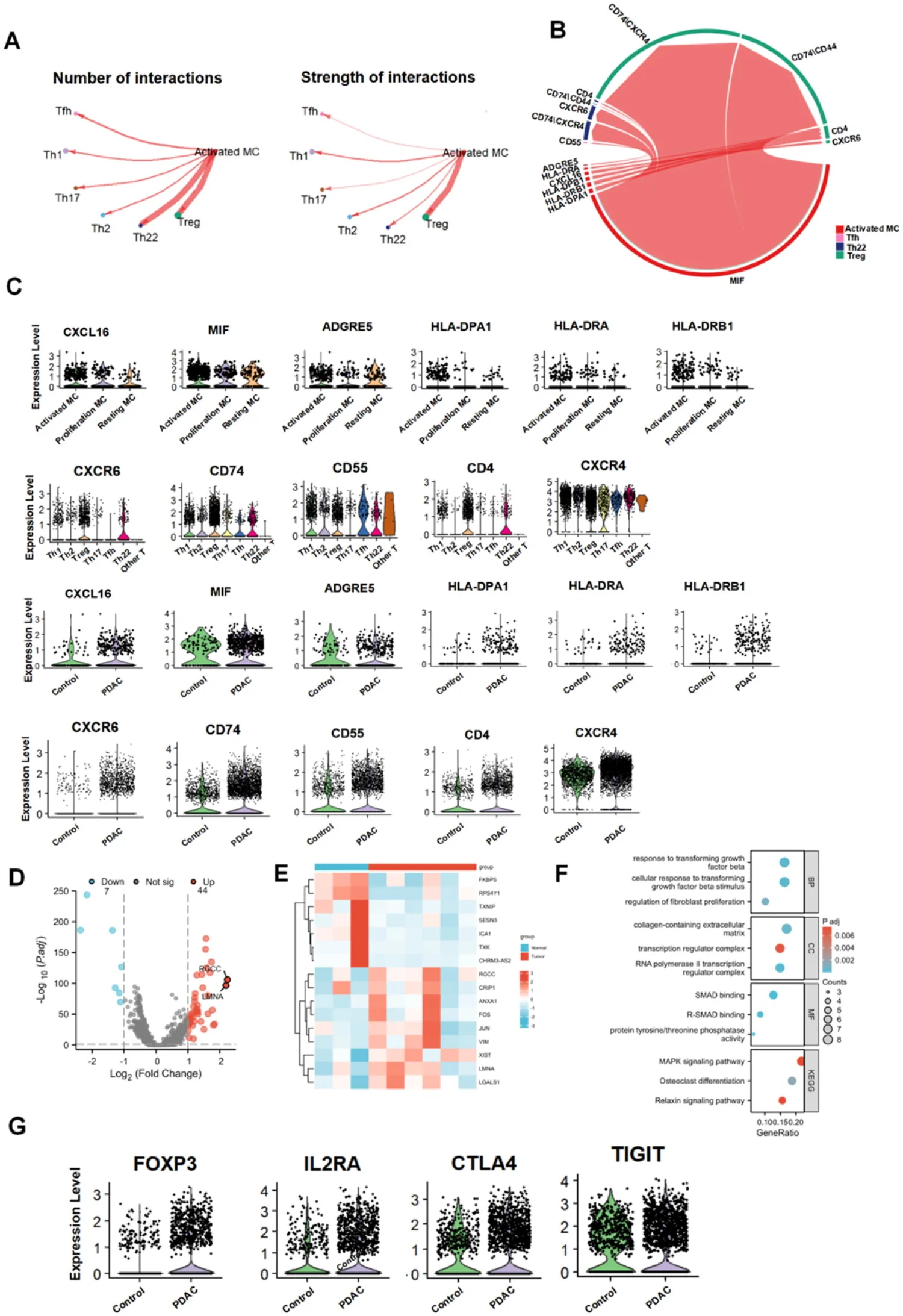
The MIF signaling axis may facilitate strong MC-Treg interactions and potentially contribute to Treg functional maturation. (A) Chord diagram generated by CellChat analysis showing the interaction strength (right) and the interaction number (left) between activated MCs and CD4^+^ T cell subtypes in PDAC. (B) Chord diagram generated by CellChat analysis showing the potential ligand-receptor interactions between activated MCs and Tfh, Th22 and Treg in PDAC. (C) Violin plots show the expression levels of potential ligand-receptor-related genes across CD4^+^ T cell subtypes, MCs subtypes, PDAC tissues, and adjacent non-tumour tissues. (D) Volcano plot displays the DEGs of Treg between PDAC tissues and adjacent non-tumour tissues. (E) Heatmap displays the DEGs of Treg between PDAC tissues and adjacent non-tumour tissues. The tissue type is indicated by the colour above the heatmap. (F) KEGG enrichment analysis of upregulated DEGs in PDAC Treg. (G) Violin plots show the expression levels of FOXP3, IL2RA, CTLA4 and TIGIT in PDAC tissues and adjacent non-tumour tissues.

To decipher how activated MCs shape CD4^+^ T cell immunity, we first mapped their communication networks with different CD4^+^ T subsets. CellChat analysis revealed that among all CD4^+^ T cells, the interactions between activated MCs and Tregs were the most prominent, exhibiting the greatest number and strength (Figure 5A). Further ligand-receptor analysis identified several high-strength connections, including MIF-CD74/CXCR4, HLA-DR/DP-CD4, CXCL16-CXCR6, ADRGE5-CD55 (Figure 5B). Further, by integrating the expression levels of ligands and receptors across cell subsets and tumour tissues, the MIF-CD74/CXCR4 signaling axis was prioritized as the most strongly predicted pathway connecting MCs and Tregs (Figure 5C). This pathway is known to promote Treg recruitment and function, which provide a mechanistic hypothesis for subsequent exploration.

We next investigated whether this MIF-mediated cellular crosstalk was associated with functional changes in Tregs. Firstly, in PDAC Tregs not only increased in abundance (Figure 4G), but also exhibited differential gene expression with 44 upregulated and 7 downregulated genes, such as downregulated FKBP5 and TXK, and upregulated RGSS and LMNA (Figure 5D,E). We additionally performed exploratory cell-level comparisons between Tregs derived from tumour and adjacent non-tumour tissues using FindMarkers. Because these comparisons did not account for the non-independence of cells originating from the same donor, the resulting expression differences are presented as descriptive, hypothesis-generating cell-state patterns rather than confirmatory donor-level differential-expression findings. KEGG and GO confirmed that PDAC Tregs were significantly enriched in pathways related to immunosuppression, cell proliferation, and tissue fibrosis (Figure 5F). Moreover, the core Treg transcription factor FOXP3, the functional receptor IL2RA (CD25), and key immune checkpoint molecules CTLA4 and TIGIT were all significantly upregulated in tumour tissues (Figure 5G). Collectively, these data computationally prioritize MIF-associated signaling as a candidate communication pathway linking activated MCs with a mature, immunosuppressive Treg state in PDAC. This association supports a testable biological model.

### 3.6. Spatial validation of MC-Treg co-localization and its clinical association

**Figure 6.**
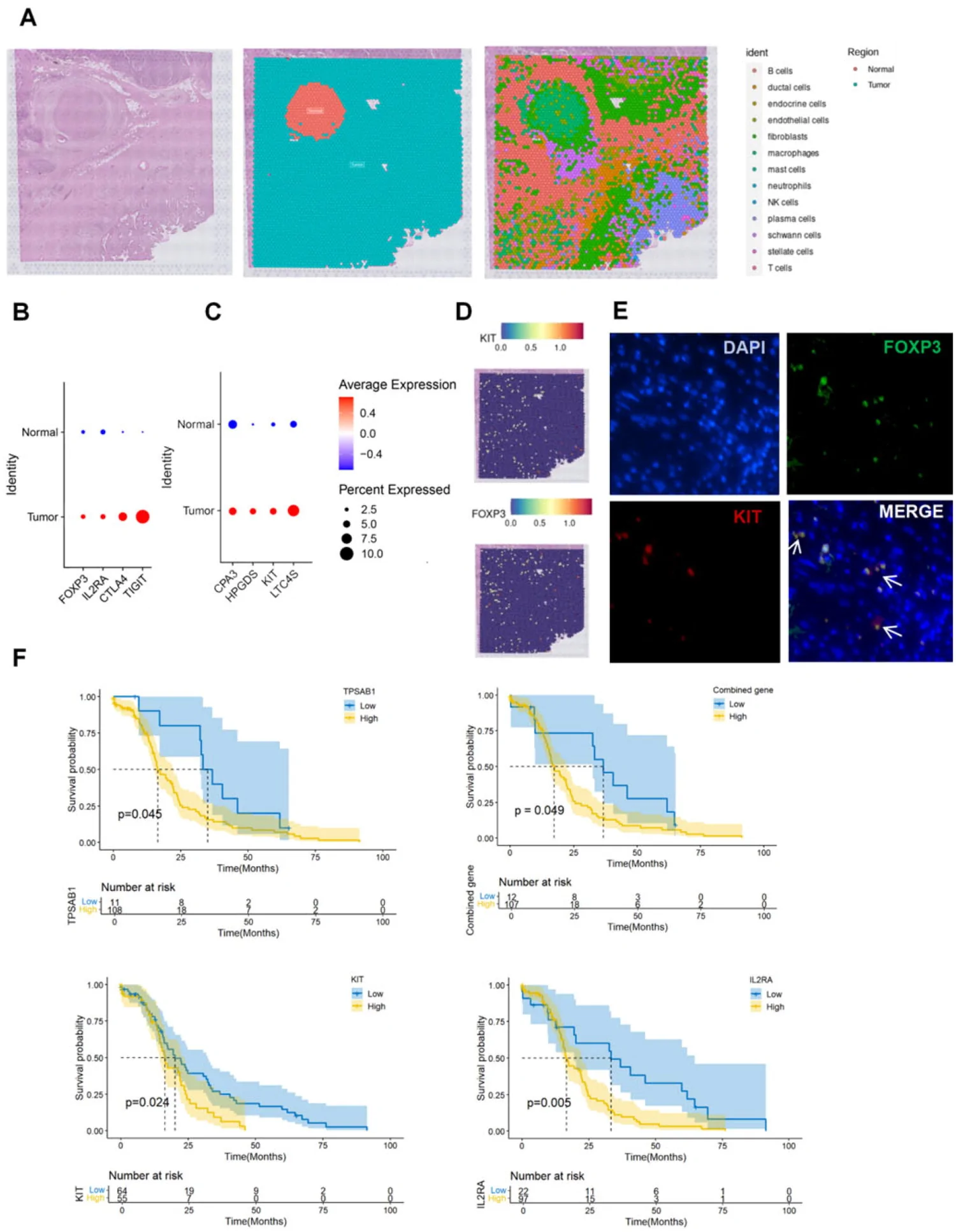
Spatial validation of MC-Treg co-localization and its clinical association. (A) Spatial plots of H&E staining (column 1), tissue regions (column 2), major cell type in a PDAC sample. (B) Dot plots display the expression of the MC signature genes in different tissue regions. (C) Dot plots display the expression of the Treg signature genes in different tissue regions. (D) Spatial plots of MCs (KIT) (up), Treg (FOXP3) (down) in a PDAC sample. € Immunofluorescence staining of human PDAC tissue. KIT (red), FOXP3 (green), DAPI (blue), in individual and merged channels are shown. Data are shown from n = 5 biological replicates. (F) Kaplan-Meier (K-M) curves show the association between the expression of the MCs-related gene (TPSAB1, KIT), Treg-related genes (IL2RA), and combined genes, and the survival prognosis of PDAC patients. *P* values were determined by the log-rank test (Mantel–Cox test).

To provide spatial and clinical substantiation for the functional MC-Treg interaction uncovered above, we first analyzed spatial transcriptomic data. The tissue was segmented into histologically annotated normal and tumour regions (Figure 6A). Initial analysis confirmed that key signature genes for MCs (including CPA3, HPGDS, KIT, and LTC4S) and Tregs (including FOXP3 and IL2RA), along with critical immune checkpoint molecules (CTLA4 and TIGIT), were significantly elevated in tumour tissues compared to normal counterparts (Figure 6B,C). Furthermore, spatial mapping showed that MC- and Treg-associated gene signatures were concurrently enriched within tumour regions, indicating overlapping spatial distribution patterns and supporting their preferential accumulation in the tumour immune microenvironment (Figure 6D). Consistent with this observation, dual immunofluorescence staining for the MC markers KIT or TPSAB1 together with the Treg marker FOXP3 revealed the presence of MCs and Tregs in close spatial proximity within tumour tissues (Figure 6E and Figure S8). Although these findings do not establish direct cellular interactions or MC-mediated Treg recruitment, they support a potential spatial association between the two cell populations. We therefore next assessed the clinical prognostic significance of the combined MC–Treg-associated signature. Survival analysis based on TCGA data revealed that high expression of a core MC signature (including TPSAB1 and KIT), the Treg marker IL2RA, and notably, a combined signature of all three, were all significant predictors of poorer overall survival in pancreatic cancer patients (Figure 6F). Collectively, these findings indicate that activated MC-Treg unit not only as a key driver of immunosuppression but also as a tangible biomarker of adverse patient outcomes.

## 4. Discussion

**Figure 7.**
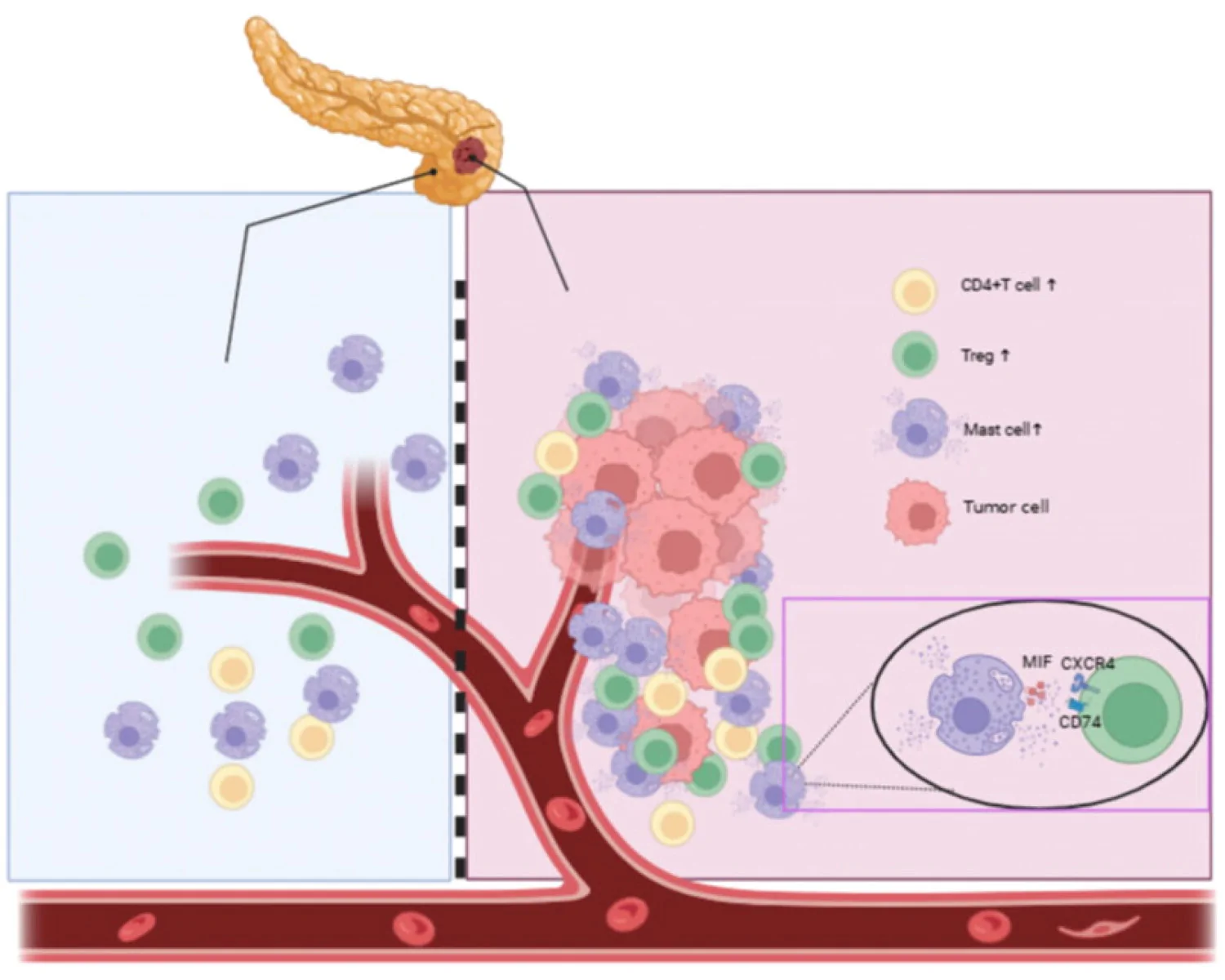
Working model: MCs as potential contributors to immunosuppression in PDAC, possibly involving recruitment and activation of Tregs.

This study systematically delineates the critical role of MCs in PDAC by integrating multi-omics data. We confirmed the specific enrichment of MCs in PDAC and resolved their high activation and functional heterogeneity. More importantly, our analyses suggest a potential pathway whereby activated MCs may contribute to immunosuppression through interactions with Tregs *via* the MIF-CD74/CXCR4 signaling axis (Figure 7). This discovery provides a new perspective for understanding PDAC’s immunosuppressive ecology and offers insights for improving immunotherapy efficacy.

First, using scRNA-seq and subsequent immunohistochemical validation, we confirmed a significant increase in MC proportion in PDAC tissues. This finding is consistent with a previous study that reported significantly increased MC infiltration in the PDAC TME [36]. Beyond mere enrichment, the scRNA-seq demonstrates that MCs in PDAC are heterogeneous, comprising resting, proliferative, and activated subtypes, and the further analyses unraveled the dynamic reprogramming of MC functional states within the PDAC TME. Pseudotime trajectory analysis indicated a differentiation bias from resting MCs towards both ‘activated’ and ‘proliferative’ fates, revealing an active process of MC ‘education’ orchestrated by the tumour. This state transition is likely driven by cytokines in the TME such as Stem Cell Factor (SCF) and IL-33 [37–39]. More importantly, this state transition is underpinned by distinct molecular programs. The activated MCs were significantly enriched in KRAS signaling, TNF-α signaling *via* NF-κB, inflammatory response and so on pathways, suggesting that their pro-tumour phenotype is likely directly instructed by the core oncogenic drivers and the pervasive inflammatory milieu of the tumour [40,41]. In essence, tumour cells not only recruit MCs but also ‘reprogram’ them into accomplices of tumour progression *via* hallmark mutations and secreted cytokines. Differently, the proliferative MCs were enriched in pathways governing growth and survival, such as IL-6-JAK-STAT3 signaling, IL-2-STAT5 signaling, PI3K-AKT-mTOR signaling and so on. This provides them with the cell-intrinsic machinery to sustain their population expansion within the harsh TME [42]. Collectively, our data paint a coherent picture: The PDAC TME actively ‘educates’ MCs beyond mere recruitment. It drives their bifurcation into two specialized states: an activated state for immunomodulation and stroma remodeling, and a proliferative state for self-renewal. Consequently, MCs become deeply embedded as pivotal components within the TME, ultimately fostering tumour growth.

Furthermore, we identified six genes—TPSAB1, TPSB2, CPA3, HPGDS, KIT, and LTC4S—as characteristic markers for PDAC MCs. These genes were consistently highly expressed across datasets and independent cohorts, suggesting their diagnostic and therapeutic potential. Notably, this signature encompasses key aspects of MC activation: the surface receptor (KIT), degranulation proteases (TPSAB1, TPSB2, CPA3), and synthases for inflammatory mediators (HPGDS, LTC4S). It depicts a highly activated phenotype, equipping MCs with the full molecular arsenal to execute pro-tumour functions [43]. These activated MCs secrete substantial amounts of proteases (tryptase, chymase), lipid mediators (prostaglandins, leukotrienes), and cytokines such as IL-13, IL-18, VEGFA, and CSF1. This diverse secretome does not only directly promote tumour angiogenesis and stromal remodeling [31], but also actively shapes an immunosuppressive microenvironment [36]. Specifically, these mediators recruit and polarize immune cells like MDSCs and TAMs, while simultaneously suppressing cytotoxic T-cell activity [44–46]. This establishes MCs as active immunoregulatory hubs within the TME, coordinating a network that fosters tumour progression.

Building on MC’s role as immunoregulatory hubs, our study delineates a candidate signaling model by which MCs sculpt the profoundly immunosuppressive T-cell landscape in PDAC. We observed a T-cell ecosystem characterized by hampered CD8^+^ T-cell activation and infiltration, countered by a CD4^+^ T-cell expansion that was significantly skewed towards Tregs. The significantly decreased Th1/Th2 and Th1/Treg ratios provide key quantitative evidence for the immunosuppressive nature of this ecosystem. This pattern typifies the dysfunctional immunity in pancreatic cancer [10,47]. Our key finding is the candidate signaling model by which MCs shape the immunosuppressive landscape. We discovered exceptionally active communication between activated MCs and Tregs, with the MIF-CD74/CXCR4 signaling axis identified as the critical bridge. MIF, a pleiotropic cytokine, has been reported to drive immunosuppression in various cancers by promoting Treg recruitment and function [48]. Our data identify activated MCs as a potential source of MIF-associated signaling and support a testable model in which this signaling may contribute to Treg accumulation and the maintenance of an immunosuppressive Treg state in PDAC. Although our analyses predict that MCs may interact with Tregs *via* the MIF–CD74/CXCR4 axis, it is important to note that these predictions are based on transcriptomic and computational analyses. We did not perform direct functional assays such as MIF neutralization, Treg chemotaxis, or co-culture experiments. Furthermore, the significant enrichment of the IL-2-STAT5 signaling pathway in activated and proliferating MCs underscores their highly activated state. The high-affinity IL-2 receptor, composed of IL2RA (CD25), IL2RB, and IL2RG, is crucial for Treg stability, function, and survival within the TME [49]. While this raised the possibility of IL-2 competition, the observed enhancement of Treg abundance argues against such a model and instead suggests that the IL-2-STAT5 activity is a hallmark of MCs that utilize IL-2-independent mechanisms to promote Tregs. This concept is complemented by interactions between HLA class II on MCs and T-cell receptors on Tregs, as well as reports that pancreatic cancer-derived exosomes can induce MCs to secrete CXCL10 for Treg recruitment [50]^.^ In summary, the predicted MIF-, HLA-II-, and CXCL10-associated interactions suggest that activated MCs may be linked to Treg enrichment and immunosuppressive activity through multiple parallel signaling pathways. These findings highlight the potential complexity of MC-Treg communication in PDAC and provide a testable model for future functional investigation. PDAC-derived Tregs not only exhibit typical immunosuppressive functions but also show significant enrichment of DEGs in pathways related to cell proliferation and tissue fibrosis. This finding thereby directly links the MC-Treg axis with two hallmark features of pancreatic cancer: immunosuppression and desmoplasia that characterizes the disease [51,52]. Consequently, we propose that MCs do not solely facilitate immune escape *via* Tregs but may also contribute to stromal remodeling through these interactions. This synthesis offers a new, integrated perspective for understanding the unique and coupled biology of the immune and stromal compartments in the PDAC microenvironment. The clinical relevance of MCs in PDAC is well-documented. Previous studies have shown that serum levels of TPSAB1 are elevated in patients [53] and that high MC infiltration at the tumour margin is associated with aggressive disease features and poor survival [54,55]. In this study, the significant prognostic power of a combined signature encompassing MC (TPSAB1, KIT) and Treg (IL2RA) genes underscores that it is their functional interplay, rather than their mere presence, that drives poor patient outcomes. This positions the integrated MC-Treg axis as a robust prognostic biomarker and a compelling multi-cellular therapeutic target in PDAC (Figure 7). However, given their paradoxical roles in tumour immunity, with MCs capable of mediating anti-tumour responses while also driving immunosuppression in specific contexts, therapeutic strategies targeting MCs must be approached with caution [20]. Our research therefore advocates for a more nuanced strategy: specifically disrupting the immunosuppressive functions of MCs, rather than achieving their complete eradication. For instance, targeted blockade of the MIF signaling axis or the selective depletion of the activated MC subpopulations identified in our study could potentially reverse the immunosuppressive tumour microenvironment (TME) while preserving the beneficial anti-tumour aspects of MC biology.

Several limitations of our study should be acknowledged. First, although we expanded our *in vitro* experiments to include additional cell lines (LAD2 MCs and BXPC3 PDAC cells), these models may still not fully capture the heterogeneity of primary cells or tumours. Second, although our analyses implicate the MIF signaling axis in MC-Treg interactions, direct functional validation of this pathway remains absent, and these findings are currently based on a single dataset. Finally, spatial transcriptomics and immunofluorescence demonstrate co‑localization of MCs and Tregs in PDAC tissues but do not provide functional evidence of their crosstalk. Future studies using functional assays will be needed to investigate the functional relevance of this interaction.

## Supplementary Material

Supplemenatry materials.docx

## Data Availability

The data that support the findings of this study are available in the Gene Expression Omnibus (GEO) database (https://www.ncbi.nlm.nih.gov/geo/) under the accession numbers GSE212966, GSE155698, GSE205049, GSE15471, and GSE235315. These data were derived from resources available in the public domain. The processed datasets generated and/or analyzed during the current study are available from the corresponding author upon reasonable request.
