# Supplementary material for: Single-cell RNA sequencing uncovers an immunosuppressive program in PDAC mast cells: tumour-driven activation, proliferation, and association with Treg recruitment
Source: Ann Med. 2026 Aug 31;58(1):2724762. doi: 10.1080/07853890.2026.2724762 (PMC13537062; doi:10.1080/07853890.2026.2724762)
Supplement: Supplementary file 1 [file IANN_A_2724762_SM7871.docx]

Fig S1

A. t-SNE visualization of cells colored by sample identity within each dataset.
B. Quality-control violin plots for single-cell RNA-sequencing data

Fig S2

A. Volcano plot displays the DEGs between actived MCs and Resting MCs.
B. Volcano plot displays the DEGs between proliferating MCs and Resting MCs.

Fig S3

A. Dot plots of marker genes for T cells subtypes.
B. Dot plots of marker genes for CD4^+^ subtypes.

Fig S4

A. MCs degranulation following co-culture with BxPC3.
B. TPSAB1 secretion level detected by ELISA following co-culture of MCs with BxPC3.
C. qRT-PCR analysis shows the expression levels of TPSAB1 in MCs after co-culture with BxPC3.
D. Flow cytometry analysis shows the cell cycle distribution of MCs after co-culture with BxPC3.
E. qRT-PCR analysis shows the expression levels of Cell cycle-related genes in MCs after co-culture with BxPC3.

Data are shown as mean ± SEM from n = 5 biological replicates. Statistical significance was determined by two-tailed Student’s t-test. *p < 0.05, **p < 0.01, ***p < 0.001.

Fig S5 Comparison of MCs proportions between ADJ and PDAC groups across three GEO datasets (GSE212966, GSE205049, and GSE155698). Scatter plots show the distribution of cell proportions in each sample, with blue indicating the ADJ group and red indicating the PDAC group. Each dot represents an individual sample. *p < 0.05, **p < 0.01.

Fig S6 Comparison of mast cell subtype proportions between ADJ and PDAC samples. Stacked bar plots show the relative proportions of three MC subtypes (Resting MC, Activated MC, and Proliferating MC) in adjacent normal tissue (ADJ1–ADJ3) and PDAC samples (PDAC1–PDAC6). Each bar represents one individual sample, and the y-axis indicates the percentage of each mast cell subtype within the total mast cell population.

Fig S7

A. The composition proportions of major immune cell subsets in each sample, including CD4⁺ T cells, CD8⁺ T cells, NKT cells, Naive T cells, and Other T cells.

B. The distribution of CD4⁺ T cell subpopulations, including Th1, Th2, Treg, Th17, Tfh, Th22, and un T cells.

Fig S8 Immunofluorescence staining of human PDAC tissue and paired NC tissue. TPSAB1 (red), FOXP3 (green), DAPI (blue), in individual and merged channels are shown. Data are shown from n = 5 biological replicates.

Supplementary Table 1 Summary of datasets used in this study

| Dataset | Accession Number | Platform | Tumor | Adjacent-Normal | Role in Analysis |
| --- | --- | --- | --- | --- | --- |
| scRNA-seq Dataset 1 | GSE212966 | 10x Genomics | 6 | 3 | Calculated the relative proportion of each cell subtype across individual samples |
| scRNA-seq Dataset 2 | GSE155698 | 10x Genomics | 14 | 3 |  |
| scRNA-seq Dataset 3 | GSE205049 | 10x Genomics | 9 | 8 |  |
| Bulk RNA-seq | GSE15471 | GPL1570 | 36 | 36 | TME cell composition |
| Bulk RNA-seq | TCGA-PAAD | Bulk RNA-seq | 179 | 171 | Expression validation and survival analysis |
| Spatial Transcriptomics | GSE235315 | GPL24676 | 7 | 0 | Spatial localization of MCs and Tregs |

Supplementary Table 2 Summary of patients used in this study

| Experiment | Paired tissues | n per assay | Patient ID | Age | Sex | Tumor stage | Differentiation |
| --- | --- | --- | --- | --- | --- | --- | --- |
| Toluidine Blue | Tumor/Normal | 5 | P001 | 70-  80 | Female | Moderately to poorly differentiated adenocarcinoma | III |
|  |  |  | P002 | 70-  80 | Male | Moderately to poorly differentiated adenocarcinoma | IB |
|  |  |  | P003 | 60-  70 | Male | Moderately to poorly differentiated adenocarcinoma | IIB |
|  |  |  | P004 | 50-  60 | Male | Moderately to poorly differentiated adenocarcinoma | IIB |
|  |  |  | P005 | 40-  50 | Female | Moderately to poorly differentiated adenocarcinoma | IIB |
| Immunofluorescence | Tumor | 5 | P001-P005 |  |  |  |  |
| qPCR | Tumor/Normal | 5 | P006 | 40-  50 | Female | Moderately differentiated adenocarcinoma | IA |
|  |  |  | P007 | 60-  70 | Male | Moderately differentiated adenocarcinoma | IIB |
|  |  |  | P008 | 60-  70 | Female | Moderately to poorly differentiated adenocarcinoma | IIB |
|  |  |  | P009 | 40-  50 | Male | Moderately to poorly differentiated adenocarcinoma | IIB |
|  |  |  | P010 | 50-  60 | Female | Moderately differentiated adenocarcinoma | IB |

Supplementary Table 3 Primer sequences

| Gene | Forward primer | Reverse primer |
| --- | --- | --- |
| Human GAPDH | TGACAACTTTGGTATCGTGGAAGG | AGGGATGATGTTCTGGAGAGCC |
| Human CDC6 | ACCTATGCAACACTCCCCATT | TGGCTAGTTCTCTTTTGCTAGGA |
| Human CCNA2 | GGATGGTAGTTTTGAGTCACCAC | CACGAGGATAGCTCTCATACTGT |
| Human CDK1 | GGATGTGCTTATGCAGGATTCC | CATGTACTGACCAGGAGGGATAG |
| Human CCNL1 | AACCCTGGTTCAAACTGCCTG | TGCAAGGTAGATGCAAGCACA |
| Human CDK2 | CCAGGAGTTACTTCTATGCCTGA | TTCATCCAGGGGAGGTACAAC |
| Human CDKN | CGATGGAACTTCGACTTTGTCA | GCACAAGGGTACAAGACAGTG |
| Human CCND3 | TACCCGCCATCCATGATCG | AGGCAGTCCACTTCAGTGC |
| Human CPA3 | AGGCACAGCTACGCAAAATAC | TGCCACAATCCGTAAAAATAGCC |
| Human LTC4S | CAGGTGAACTGCAGCGAGTA | GACTAGGATGCGGAAGCGAG |
| Human KIT | ACTTGAGGTTTATTCCTGACCCC | GCAGACAGAGCCGATGGTAG |
| Human HPGDS | ACCAGAGCCTAGCAATAGCAA | AGAGTGTCCACAATAGCATCAAC |
| Human TPSAB1 | GCAGGTGAGCCTGAGAGTC | CTGGGCGGTGTAGAACTGTG |
| Human TPSB2 | GAGAGTCCGCGACCGATACT | CTGGGCGGTGTAGAACTGTG |

**MERGE**
